# Electrochromic Self-Electrostabilized Polypyrrole Films Doped with Surfactant and Azo Dye

**DOI:** 10.3390/polym11111757

**Published:** 2019-10-25

**Authors:** Maryam Bayat, Hossein Izadan, Brenda G. Molina, Margarita Sánchez, Sara Santiago, Dariush Semnani, Mohammad Dinari, Gonzalo Guirado, Francesc Estrany, Carlos Alemán

**Affiliations:** 1 Department of Textile Engineering, Isfahan University of Technology, Isfahan 84156-83111, Iran; maryam.bayat@tx.iut.ac.ir (M.B.); d_semnani@cc.iut.ac.ir (D.S.); 2 Departament d’Enginyeria Química, EEBE, Universitat Politècnica de Catalunya, C/ Eduard Maristany 10-14, Ed. I2, 08019 Barcelona, Spain; brenda.guadalupe.molina@upc.edu (B.G.M.); margarita.sanchez@upc.edu (M.S.); francesc.estrany@upc.edu (F.E.); 3 Barcelona Research Center for Multiscale Science and Engineering, Universitat Politècnica de Catalunya, Eduard Maristany 10-14, 08019 Barcelona, Spain; 4 Departament de Química, Universitat Autònoma de Barcelona, 08193 Barcelona, Cerdanyola del Vallès, Spain; sara.santiago@uab.cat (S.S.); Gonzalo.Guirado@uab.cat (G.G.); 5 Department of Chemistry, Isfahan University of Technology, Isfahan 84156-83111, Iran; dinari@cc.iut.ac.ir

**Keywords:** conducting polymer, electrochromic properties, optoelectronics, sodium dodecyl sulfate, spectroelectrochemistry

## Abstract

Two azo dyes, acid red 1 (AR1) and acid red 18 (AR18), were used alone or in combination with sodium dodecyl sulfate (SDS) for the electropolymerization of a pyrrole monomer. Polypyrrole (PPy) showed higher redox capacity when SDS and AR18 were used simultaneously as dopant agents (PPy/AR18-SDS) than when the conducting polymer was produced in the presence of SDS, AR18, AR1, or an AR1/SDS mixture. Moreover, PPy/AR18-SDS is a self-stabilizing material that exhibits increasing electrochemical activity with the number of oxidation–reduction cycles. A mechanism supported by scanning electron microscopy and X-ray diffraction structural observations was proposed to explain the synergy between the SDS surfactant and the AR18 dye. On the other hand, the Bordeaux red color of PPy/AR18-SDS, which exhibits an optical band gap of 1.9 eV, rapidly changed to orange-yellow and blue colors when films were reduced and oxidized, respectively, by applying linear or step potential ramps. Overall, the results indicate that the synergistic utilization of AR18 and SDS as dopant agents in the same polymerization reaction is a very successful and advantageous strategy for the preparation of PPy films with cutting-edge electrochemical and electrochromic properties.

## 1. Introduction

In the last few decades, intrinsically conducting polymers (ICPs) emerged as efficient materials for electronic applications, opening up the era of plastic electronics. Among heterocyclic ICPs, polypyrrole (PPy) captured the attention of the scientific community since its interesting characteristics (e.g., conductivity, environmental stability, mechanical properties, biocompatibility, and high yield in redox processes) can be easily tuned with the dopant agent [1,2,3,4,5]. Due to these advantageous properties, PPy was used in many technological and biomedical applications, for example, electrodes for batteries [6,7] and supercapacitors [8,9], bioactive platforms for cell adhesion [10,11] and biosensing [12,13,14], neural interfaces [15,16], and electrochromic devices [17,18,19].

The incorporation of organic dyes with reversible optoelectronic properties into the PPy backbone is a potential approach to achieve enhanced optical contrast and color modulation [18,19,20,21,22,23,24,25,26,27,28,29]. Although many organic dyes have poor solubility in water due to strong π–π interaction and planar structure, acidic dyes with solubility-enhancing groups, such as sulfonate groups, can be used as dopant agents, acting as charged species that promote the PPy conductivity. Thus, the large conjugated system of dyes can exert electronic interaction with the π-system of the ICP, altering the electronic properties of PPy [24].

The influence of some azo dyes (i.e., those bearing the R–N=N–R’ functional group, where R and R’ are usually aryl groups), for example, Remazol Black B [25], on the electrochromic properties of PPy was studied due to their low cost. Compared to common dopant ions, these organic molecules, which are frequently used to treat textiles and leather articles, provide easy mass transport at the interface and charge transfer in the bulk, improving the electrochromic properties of the ICP. Another approach consisted of the incorporation of azo dyes into the ICP backbone [26,27,28]. However, although monomers undergo *trans*–*cis* photoisomerization upon irradiation, in some cases, the electrochromic properties of polymer films can be limited by the rigid conjugated backbone [30].

On the other hand, the influence of surfactants in the preparation and properties of ICPs is well known [29,30,31,32,33,34,35,36,37,38]. In general, it is observed that ICPs prepared with anionic surfactants, for example, sodium dodecyl sulfate (SDS), have higher conductivity and electrochemical activity compared to those prepared using non-ionic and cationic surfactants, due to the role of anionic surfactants as a dopant agent. Moreover, the preparation of ICPs using anionic micelle solutions shows an enhanced polymerization rate because of molecular interactions between anionic surfactants and the formed radical cations.

**Scheme 1:**
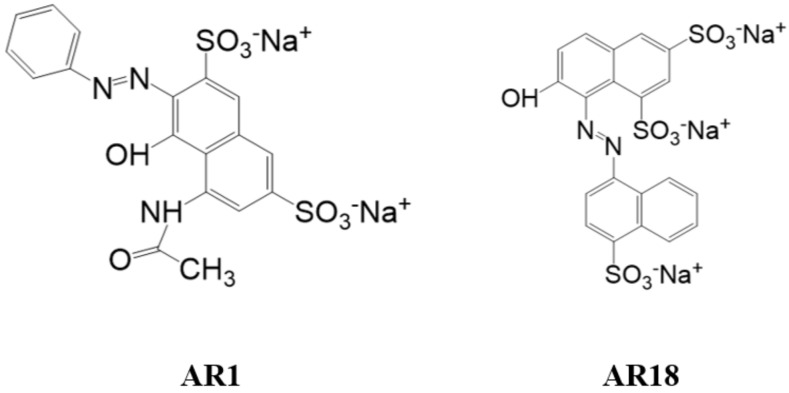
Chemical structures of AR1 and AR18.

In spite of the advantages provided by azo dyes and surfactants, when they are used separately, the synergistic effect associated with their simultaneous utilization as dopant agents remains to be tested. In this work, we examined the electrochemical, structural, and optical properties of PPy doped with a synthetic dye and a surfactant together (PPy/dye-surfactant). Results were compared with those obtained when the same ICP was doped with each of these species separately (PPy/dye and PPy/surfactant). For this purpose, SDS was chosen as a surfactant, while two azo dyes currently used in the textile industries (Scheme 1), named acid red 1 (AR1) and acid red 18 (AR18), were considered. Due to its stability and multiple colors, the system that combines PPy with both AR18 and SDS is a promising material for application in future electrochromic devices, for example, e-paper, sunroofs, visors, smart windows and walls, and active optical filters.

## 2. Materials and Methods

### 2.1. Materials

Pyrrole (Py) from Aldrich was doubly distilled and stored in a refrigerator and protected from light before use. SDS and anhydrous lithium perchlorate (LiClO_4_) of analytical reagent grade were purchased from Sigma-Aldrich. AR1 from Sigma-Aldrich and AR18 from Panreac were used as received. All solutions were prepared using Milli-Q water.

### 2.2. Synthesis

PPy films doped with ClO_4_^−^ (control), AR1, AR18, and/or SDS, hereafter denoted PPy/dopant, were prepared using different electrochemical techniques: (i) chronopotentiometry (CP) by applying currents of 1.5 (ClO_4_^−^ and SDS) and 1.0 mA/cm^2^ (SDS, AR1, and AR18) for 200 s; (ii) cyclic voltammetry (CV) applying three scans with a potential interval between −0.30 V (initial and final potential) and 1.3 V (reversal potential) at a sweep rate 30 mV/s; and (iii) chronoamperometry (CA) by applying a constant potential of 1.0 V for 300 s. Unfortunately, it was not possible to generate homogeneous PPy/ClO_4_^−^ films using CP at 1.0 mA/cm^2^ nor homogeneous PPy/AR18-SDS, PPy/AR18, PPy/AR1-SDS, and PPy/AR1 films at 1.5 mA/cm^2^. Furthermore, good PPy/SDS films were achieved at both 1.5 and 1.0 mA/cm^2^. In spite of these differences, the thickness (*L*) of all studied films was very similar, as shown in Table 1.

The different PPy films were prepared using a three-electrode one-compartment cell under nitrogen atmosphere (99.995% in purity) at 25 °C. In all cases, ITO sheets of 1 × 1.5 cm^2^ area were used as a working electrode, bare steel AISI 316L sheets of 1.5 × 2 cm^2^ area were used as a counter electrode, and an Ag|AgCl electrode containing a KCl-saturated aqueous solution (E^0^ = 0.222 V at 25 °C) was used as the reference electrode. The cell was filled with 10 mL of a 0.1 M Py aqueous solution. The different PPy/dopant systems synthesized in this work are summarized in Table 1, which also lists the concentration of dopant agent(s) used in each case.

### 2.3. Characterization

Electrochemical assays were conducted on an Autolab PGSTAT302N (Ecochimie, the Netherlands) potentiostat–galvanostat equipped with NOVA software and using a conventional three-electrode system. Electrochemical characterization was performed by CV using a 0.1 M LiClO_4_ aqueous solution. The potential range was chosen between −1.00 V (initial and final potential) and +1.00 V (reversal potential) vs. Ag/AgCl, and the scan rate was 100 mV/s unless another value is explicitly indicated.

The redox capacity and the electrochemical stability (electrostability) of the prepared films were determined by cyclic voltammetry. The redox capacity, which refers to the charge storage ability, was defined as the maximum voltammetric stored charge per surface unit (Q, in C/cm^2^) in the second oxidation–reduction cycle. The electrostability was evaluated as the variation of Q with consecutive oxidation–reduction cycles. Thus, the redox capacity increases with the similarity between the anodic and cathodic areas of the first control voltammogram, whereas the electrostability decreases with the oxidation and reduction areas of consecutive control voltammograms.

The electrical resistivity was measured using the sheet resistance method from films synthesized on steel electrodes of 3 cm^2^ area.

Optical parameters were determined using spectroelectrochemical techniques. A VSP100 potentiostat model was coupled to a L12090 Hamamatsu spectrophotometer; both instruments were controlled and synchronized using EC-Lab V9.51 and Biokine 32 V. 4.46 software. The spectroelectrochemical characterization of the samples was carried out using indium tin oxide ITO sheets as the working electrode, which were previously modified by electrodeposited PPy films. A Pt wire was used as the counter electrode, and a saturated calomel electrode (SCE) was used as the reference electrode. Finally, an aqueous solution (0.1 M LiClO_4_) was used as the supporting electrolyte. The spectroelectrochemical experiments were performed using controlled potential electrolysis; hence, a constant reduction potential was firstly applied, followed by a constant oxidation potential. The corresponding ultraviolet–visible light (UV–Vis) absorbance spectra were recorded simultaneously. From the optical data, different optical parameters were calculated.

Film thickness and roughness measurements were carried out using a Dektak 150 stylus profilometer (Veeco, Plainview, NY, USA). Different scratches were intentionally caused on the films and measured to allow statistical analysis of data. At least 18 independent measurements were performed for three samples of each examined condition. Imaging of the films was conducted using the following optimized settings: tip radius = 12.5 µm; stylus force = 3.0 mg; scan length = 0.5 mm.

Morphological analysis of the films was conducted by scanning electron microscopy. A Focus Ion Beam Zeiss Neon 40 instrument (Carl Zeiss, Germany) equipped with an energy-dispersive X-ray (EDX) spectroscopy system, operating at 5 kV, was used.

Wide-angle X-ray scattering (WAXS) diagrams were recorded in vacuum at room temperature using calcite as a calibration standard. A modified Statton Warhus (Wilmngton, DE, USA) camera and NiYCu radiation (λ = 0.1542 nm) were used. Measurements were performed in a 2*θ* range of 5–80°, measurement steps of 0.02°, and a scan speed of 2 s.

Absorption spectra were obtained with a Shimadzu 3600 spectrophotometer equipped with a tungsten halogen visible source, a deuterium arc UV source, a photomultiplier tube UV–Vis detector, an In–Ga–As photodiode, and cooled PbS photocell near-infrared (NIR) detectors. Spectra were recorded in the absorbance mode using the integrating sphere accessory (model ISR-3100), with a wavelength range of 300–800 nm. The interior of the integrating sphere was coated with a highly diffuse BaO reflectance standard. Single-scan spectra were obtained at a scan speed of 60 nm·min^−1^ with a bandwidth of 2 nm using the UVProbe 2.31 software. The optical ε_g_ was derived from the UV–Vis spectra using a previously described procedure [39].

## 3. Results and Discussion

### 3.1. Electrochemical Behavior

**Figure 1:**
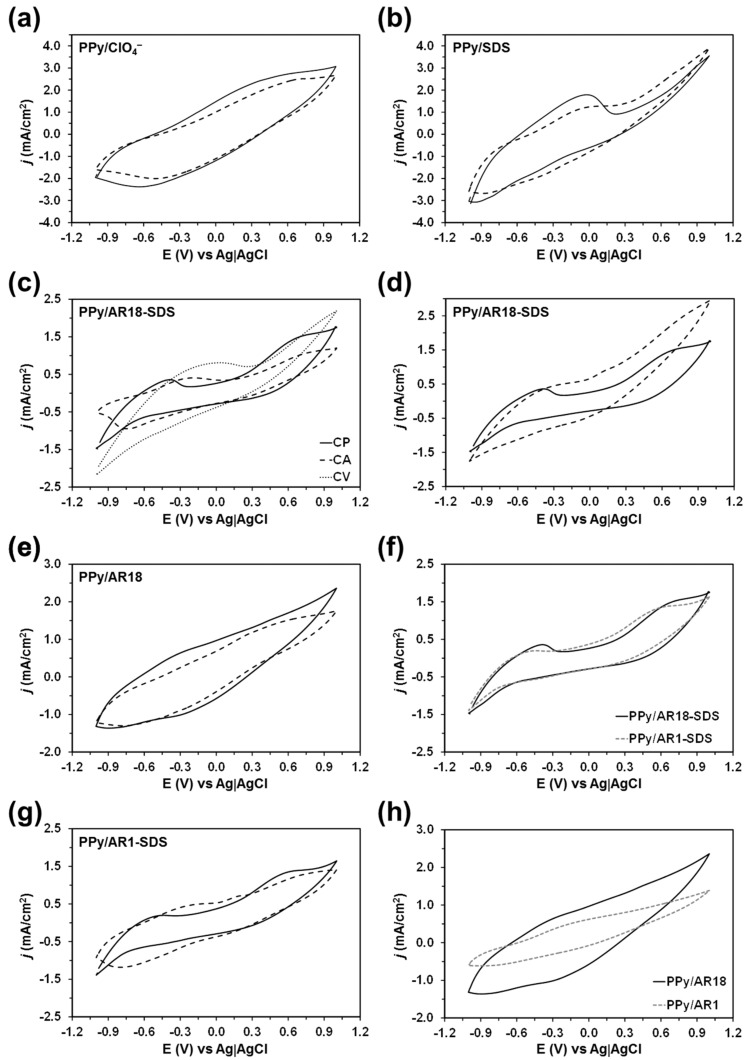
Cyclic voltammograms for (**a**) polypyrrole (PPy)/ClO_4_^−^ (second and 25th redox cycles), (**b**) PPy/ sodium dodecyl sulfate (SDS) (second and 25th redox cycles), (**c**) PPy/AR18-SDS (prepared using chronopotentiometry (CP), chronoamperometry (CA), and cyclic voltammetry (CV)), (**d**) PPy/AR18-SDS (second and 25th redox cycles), (**e**) PPy/AR18 (second and 25th redox cycles), (**f**) PPy/AR18-SDS and PPy/AR1-SDS (second redox cycle), (**g**) PPy/AR1-SDS (second and 25th redox cycles), and (**h**) PPy/AR18 and PPy/AR1 (second redox cycle) films. Voltammograms were recorded using a 0.1 M LiClO_4_ aqueous solution. Initial and final potential: −1.00 V; reversal potential: +1.00 V; scan rate: 100 mV/s.

The cyclic voltammetric curves recorded in a 0.1 M LiClO_4_ aqueous solution for PPy films prepared using ClO_4_^−^ and SDS as dopant agents (PPy/ClO_4_^−^ and PPy/SDS, respectively, in Table 1) are displayed in Figure 1a,b, respectively. Both the oxidation peaks and the variation of the current density along the scanned potential were more pronounced for PPy/SDS than for PPy/ClO_4_^−^. These differences were attributed to the electrocatalytic effect of SDS on the anodic polymerization of Py, as previously reported [40,41], whereas ClO_4_^−^ behaved as a simple dopant anion. Thus, SDS decreased the Py oxidation potential due to the formation of strong electrostatic interactions between the dodecyl sulfate anions and the formed radical cations. Also, the local concentration of Py monomer at the electrode/electrolyte interface was expected to be enhanced in the presence of SDS. These effects may influence different stages of the polymerization process, such as the electron transfer, the transport of the monomer toward the electrode and the reaction kinetics, the solubilization of reaction products such as oligomers, and their loss into the solution.

A comparison of the cathodic and anodic areas in the voltammetric curves obtained after two and 25 consecutive oxidation-reduction cycles indicates that the electrochemical activity of PPy/ClO_4_^−^ decreased by 16% after 25 cycles, while that of PPy/SDS increased by 4%. This is reflected in Table 2, which summarizes the variation of the electrochemical activity with the number of cycles (i.e., the electrochemical stability). Moreover, the redox capacity was initially 14% higher for PPy/ClO_4_^−^ than for PPy/SDS; however, after 25 cycles, the redox capacity was 17% higher for PPy/SDS than for PPy/ClO_4_^−^. These results indicate that, initially, the entrance of dopant anions into the PPy matrix upon oxidation and the escape of ions to the electrolyte solution upon reduction processes were greater for PPy/ClO_4_^−^ than PPy/SDS. However, the reduction of the redox capacity with the number of cycles reflects that the structure of PPy/ClO_4_^−^ was altered by such redox processes, hindering the exchange of ClO_4_^−^ ions between the PPy matrix and the electrolyte solution and explaining the loss of electrochemical stability. In contrast, the structure of the PPy matrix remained unaffected for PPy/SDS, which was attributed to the intercalation of the SDS molecules between the polymer chains (see below). This facilitated the access and escape of dopant ions and preserved the structure of the PPy matrix, which explains not only the increment of redox capacity with the number of cycles but also the fact that, after 25 cycles, the redox capacity was higher for PPy/SDS than for PPy/ClO_4_^–^. Moreover, the color of PPy/SDS films (see below) changed from yellow to blue upon oxidation and reduction, respectively, while PPy/ClO_4_^−^ control films apparently did not experience any color change.

Figure 1c compares the cyclic voltammograms recorded for PPy/AR18-SDS films, which were prepared by CP, CV, and CA (see Section 2) using both AR18 and SDS as dopant agents. As can be seen, oxidation and reduction peaks were not only better defined but also more resolved (i.e., more separated) for the film prepared using CP than for those obtained using CV and CA, indicating that the structure of the PPy chains was more homogeneous when anodic polymerization was conducted using the former method. More specifically, two oxidation peaks were detected at −0.4 and +0.7 V, which were interpreted as the formation of polarons and bipolarons, respectively, in the PPy chains [42]. In addition, two reduction peaks were identified in the cathodic scanning at −0.8 and +0.2 V, reflecting the presence of redox pairs in the recorded potential range. According to these results, the CP method was hereafter selected to prepare all azo dye-containing PPy films.

Voltammetric curves obtained for PPy/AR18-SDS films prepared by CP, which are shown in Figure 1d, indicate that the electrochemical activity increased 29% after 25 consecutive oxidation–reduction cycles (Table 2). Accordingly, PPy/AR18-SDS acts as a self-electrostabilized material, which is an unusual behavior within the context of ICPs. It is worth noting that the electrochemical activity is related to the access and escape of dopant ions during oxidation and reduction processes, respectively. These processes, which involve a change in the composition of the polymeric matrix, are usually associated with the structural expansion and contraction of the conducting polymer film, inducing its degradation (i.e., loss of redox capacity with increasing number of redox cycles). In the case of PPy/AR18-SDS films, the presence of intercalated SDS molecules (see below) facilitated the access and escape of dopant ions, preserving and stabilizing the structure of the PPy matrix. Despite the increment of the anodic and cathodic areas with the number of cycles, it should be mentioned that the oxidation and reduction peaks become less defined and resolved. Moreover, a comparison with PPy/SDS (Figure 1b) reveals that the addition of a small amount of AR18 (0.5 mM) as dopant agent resulted in a significant increment of the redox capacity, which was higher for PPy/AR18-SDS than for PPy/SDS by 43% and 52% after two and 25 redox cycles, respectively. This observation is supported by the fact that the surface morphology of PPy/AR18-SDS remained unaltered after 100 redox cycles (not shown), while, instead, drastic compaction was observed for simply doped films (i.e., without intercalated detergent molecules) [43].

A comparison of the voltammetric curves of PPy/AR18, which are shown in Figure 1e, with those of PPy/AR18-SDS (Figure 1d) indicates that the elimination of SDS resulted in a significant change. Thus, the oxidation and reduction peaks were much less pronounced for the former than for the latter. Furthermore, PPy/AR18 is not as a self-electrostabilized material since its electrochemical stability decreased by 16% after 25 consecutive redox cycles (Table 2). Accordingly, the electrochemical behavior of PPy/AR18 and PPy/AR18-SDS resembled that of PPy/ClO_4_^−^ and PPy/SDS, respectively, indicating that AR18 behaves as a simple dopant ion, similar to ClO_4_^−^, but it does not show the electrocatalytic effects associated with SDS. It is worth noting that, although the redox capacity in the second cycle increased with the film thickness (Figure 1 and Table 1), the synergy between the dye and the surfactant broke this tendency, as shown in Figure 1 and Table 2. Thus, the PPy/AR18-SDS is not only more electrostable but also thinner (Table 1 and Table 2) than PPy/AR-18 and PPy/SDS.

Figure 1f–h summarize the results obtained for PPy/AR1-SDS and PPy/AR1. A comparison of the voltammetric curves recorded for the second cycle indicates the electrochemical activity of PPy/AR1-SDS was similar to that of PPy/AR18-SDS (Figure 1f). In addition, the redox capacity of PPy/AR1-SDS increased 16% after 25 redox cycles (Figure 1g and Table 2), evidencing that this is a self-stabilized material. In spite of this, Figure 1h reflects that the behavior as a dopant agent was much weaker for AR1 than for AR18 (i.e., the electrochemical activity of PPy/AR1 was lower than that of PPy/AR18 by 36%). It is worth noting that the number of charged sulfonate groups and the average separation between them were higher for AR18 than for AR1, which improved the mass–charge transfer at the interface and had a positive effect in the electrochemical behavior of the produced film. According to these results, the rest of this work focused on PPy/AR18-SDS and, for the sake of completeness, on PPy/SDS and PPy/AR18 controls, all prepared by CP.

As shown in Table 2, the Coulombic efficiency of the prepared films, which is defined as the ratio between the oxidation and reduction charges (Q_ox_/Q_red_), was higher when the surfactant and dye acted synergistically, independently of the dye. Moreover, this effect increased with the number of redox cycles. Additionally, the Coulombic efficiency after 25 cycles was higher for PPy/SDS than for PPy/ClO_4_^−^, which is fully consistent with the corresponding variations of electrochemical activity.

**Figure 2:**
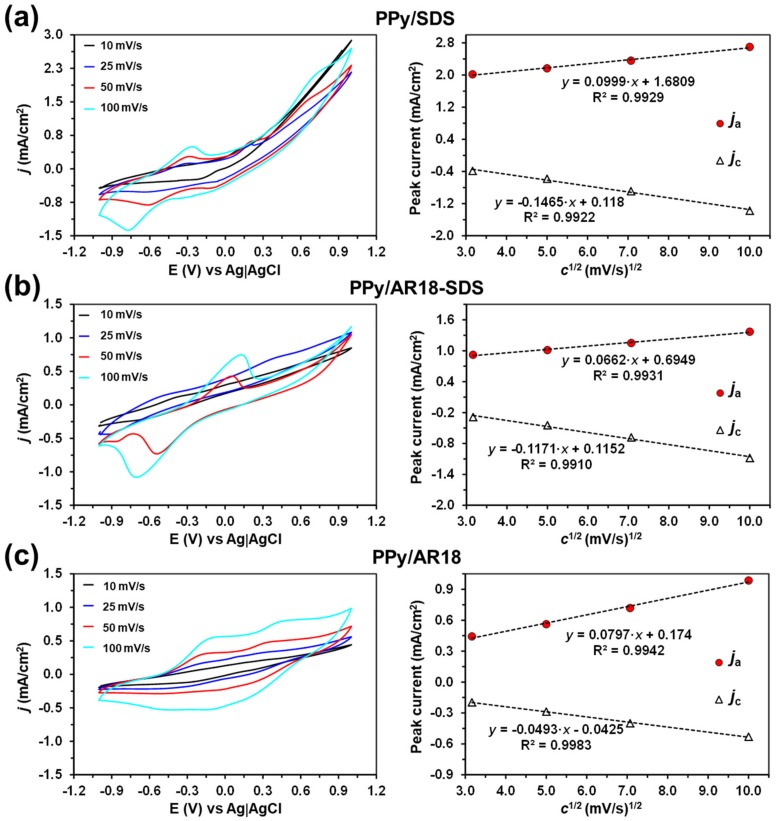
Voltammograms in 0.1 M LiClO_4_ aqueous solution at different scan rates (**left**), and dependence of the anodic and cathodic peak currents (*j*_a_ and *j*_c_, respectively) on the square root of the scan rate, *c*^1/2^ (**right**). Initial and final potential: −1.00 V; reversal potential: +1.00 V.

Figure 2 (left side) shows the voltammograms of PPy/SDS, PPy/AR18-SDS, and PPy/AR18 performed at scan rates (*c*) of 10, 25, 50, and 100 mV/s. Redox peaks, which correspond to the oxidation and reduction processes of PPy, were clearly observed, independently of the scan rate. Furthermore, as the scan rate was increased, the anodic peak shifted toward more positive potentials while the cathodic peak shifted toward more negative potentials. In addition, by increasing the scan rate, the cathodic peaks became progressively broader, suggesting that the cations and anions were no longer be able to participate completely in the doping/dedoping process at high sweep rates [44]. The logarithm of the peak current (mA/cm^2^) varied linearly as a function of the logarithm of scan rate (mV/s) with correlation coefficients higher than 0.990 in all cases (not shown). These features and the slopes, which ranged from 0.7 to 0.9, confirmed a well-adhered polymer film and a redox process partially controlled by diffusion. The linear relationships of the square root of scan rate (*c*^1/2^) with the anodic and cathodic peak currents (*j*_a_ and *j*_c_, respectively) are shown in Figure 2 (right side) for PPy/SDS, PPy/AR18-SDS, and PPy/AR18, indicating that these were typical diffusion controlled processes.

**Figure 3:**
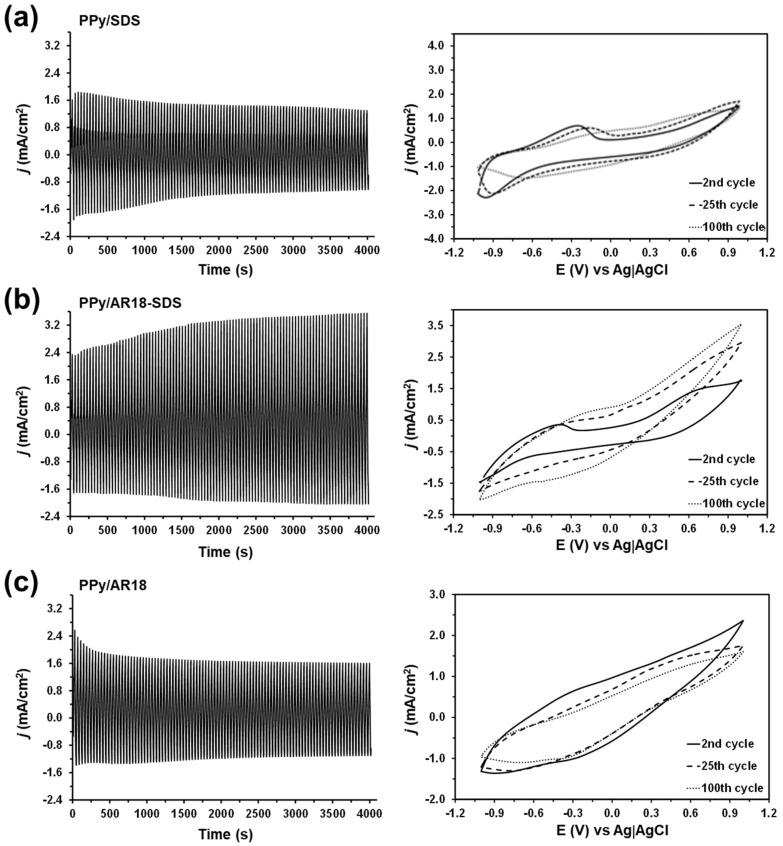
Variation of the current density versus the time (left) recorded by applying 100 consecutive oxidation–reduction cycles to (**a**) PPy/SDS, (**b**) PPy/AR18-SDS, and (**c**) PPy/AR18. Cyclic voltammograms obtained for second, 25th, and 100th cycles for the three systems are also displayed (right). Initial and final potential: −1.00 V; reversal potential: +1.00 V; scan rate: 100 mV/s.

To better understand the electrochemical stability of PPy/SDS, PPy/AR18-SDS, and PPy/AR18, 100 consecutive oxidation–reduction cycles in the potential interval from −1.0 to +1.0 V were applied at a scan rate of 100 mV/s. Figure 3 displays the variation of the current density versus the time, as well as the voltammetric curves obtained for the second, 25th, and 100th cycles for the three systems. As can be seen, the current density decreased as time increased for PPy/AR18. The loss of electrochemical activity with the increasing number of redox cycles is the expected behavior not only for PPy but also for the vast majority of ICPs. In contrast, the current density increased with time for PPy/AR18-SDS, indicating that the self-electrostabilizing behavior of this material was maintained not only for the first 25 cycles, as shown in Figure 1, but for a large number of cycles. Thus, the electrochemical activity of PPy/AR18-SDS grew 29% and 59% after 25 and 100 consecutive redox cycles, respectively. Although other self-electrostabilized materials were reported in the literature, for example, grafted copolymers made of an all-conjugated polythiophene backbone and well-defined poly(ethylene glycol) (PEG) side chains [45], this effect was maintained only for a few cycles (~10).

**Figure 4:**
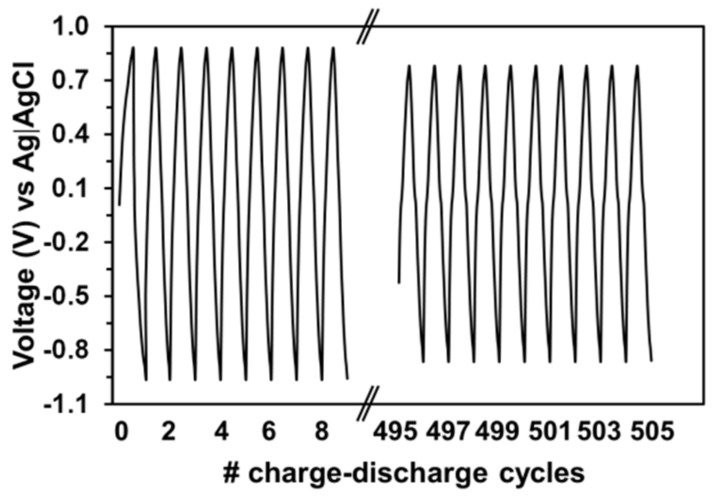
Galvanostatic charge–discharge curves for PPy/AR18-SDS recorded by applying an intensity of 0.5 A for 10 s, followed by −0.5 A for an additional 10 s. A total of 505 cycles were applied, with the graphic reporting the first few and the last few.

The stability of PPy/AR18-SDS was further investigated using galvanostatic charge–discharge cycles. Cycles were run applying a current of +0.5 mA for 10 s followed by a current of −0.5 mA for 10 s (i.e., 20 s per cycle). Results, which are shown in Figure 4, indicate that, in the second cycle, the voltage varied between 0.88 (+0.5 mV) and −0.96 V (−0.5 mA). This potential window, 1.84 V, only dropped 0.20 V after 505 cycles (i.e., the voltage varied between 0.78 and −0.86 V), reflecting the stability of PPy/AR18-SDS against galvanostatic assays.

On the other hand, the electrical resistivity was determined for PPy/SDS, PPy/AR18-SDS, and PPy/AR18 films deposited onto steel electrodes of 3 cm^2^ area. The electrical resistivity varied between 1.0 kΩ and 1.6 kΩ, comparable to the values reported for PPy electrosynthesized in aqueous micellar solutions [36]. Accordingly, the simultaneous incorporation of SDS and AR18 into the polymeric matrix had a lower effect on the electrical response than on the electrochemical one.

Electrochemical results obtained for PPy/AR18-SDS indicate that the addition of SDS to the polymerization medium is a very successful and advantageous strategy for the preparation of azo-containing PPy films. The synergy that results from combining SDS and AR18 dopant agents (i.e., their negatively charged sulfonate groups neutralize the positive charges of PPy) was attributed to the effect of their medium size combined with their flexibility or rigidity. Thus, SDS molecules are flexible due to the pending dodecyl chain, while the AR18 molecules are very rigid because of the fused aromatic rings attached to the azo functionality. Accordingly, during the polymerization process, it is expected that flexible SDS molecules organize more easily between the growing PPy chains than rigid AR18 molecules. However, the molecular size of SDS is large enough to promote a more open structure in the ICP than conventional doping agents, such as ClO_4_^−^ or Cl^−^ anions. The SDS-induced separation between PPy chains favors the ionic mobility in the interfacial zone and, therefore, enhances the redox capacity and, especially, the electrostability, as demonstrated above. Most importantly, this SDS-induced open structure allows the entry of rigid AR18 molecules as a dopant agent in a greater proportion than that observed in the absence of SDS, favoring a polymer structure with a higher doping level. It is worth noting that the electrochemical results discussed in Figure 1, Figure 2, Figure 3 and Figure 4 support this simple hypothesis, which is also consistent with SEM observations (see below).

### 3.2. Structural Characterization

The thickness (*L*) and root-mean-square roughness (R_q_) of PPy/SDS, PPy/AR18-SDS, and PPy/AR18 films are listed in Table 3. In comparison to PPy/SDS, the incorporation of AR18 caused an enlargement of the film thickness (27% and 60% for PPy/SDS-AR18 and PPy/AR18, respectively). Moreover, the dye induced a very large change in the surface R_q_, which underwent a considerable increment (66%) and reduction (79%) for PPy/AR18-SDS and PPy/AR18, respectively. Although these topographic differences are apparently contradictory, morphological analyses of these films provide an understanding of such observations.

**Figure 5:**
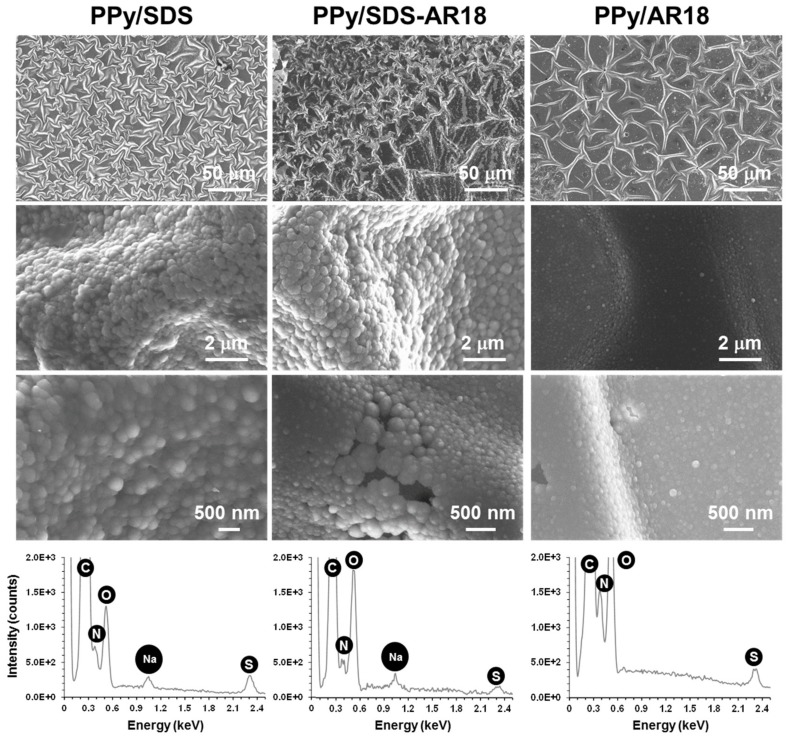
SEM micrographs at different magnifications and representative energy-dispersive X-ray (EDX) spectra of PPy/SDS (**left**), PPy/AR18-SDS (**center**), and PPy/AR18 (**right**).

Representative SEM micrographs of PPy/SDS, PPy/AR18-SDS, and PPy/AR18 are compared in Figure 5. As it can be seen, low-magnification images of PPy/SDS indicate that the polymerization of Py in the presence of surfactant induced the formation of multiple, relatively prominent and well-localized folds homogeneously distributed onto the surface. Higher-magnification images evidence that PPy maintained the typical globular or nodular morphology frequently reported for this CP. The constituent spherical PPy particles, which were of about 350–450 nm diameter, were efficiently packed, filling the space on the ITO electrode.

Substitution of SDS by AR18 resulted in drastic morphological changes. More specifically, the folds identified in PPy/AR18, which were much less abundant than in PPy/SDS, presented a very smooth surface. Indeed, the very compact and globular microstructure of these folds was formed by very small particles of a few tenths of nanometers in diameter, which explain the drastic reduction of the surface roughness with respect to PPy/SDS. The surface of PPy/AR18-SDS can be described as a hierarchical structure that combines the characteristics of PPy/SDS and PPy/AR18 globular particles. Thus, folds were pronounced but much less defined in PPy/AR18-SDS than in the other films, which was attributed to the presence of spherical particles of two well-differentiated sizes. Thus, large particles like those found in PPy/SDS coexisted with very small particles like those identified for PPy/AR18, which formed a less compact surface leaving inter-particle empty spaces as those displayed in the highest-magnification micrograph displayed in Figure 5.

SEM micrographs displayed in Figure 5 support the hypothetical mechanism used to explain the synergy between SDS and AR18 dopant agents. Thus, the larger globular particles found in PPy/SDS and PPy/AR18-SDS resulted in more opened interfacial surfaces than the small globules detected for PPy/AR18. On the other hand, local semi-quantitative elemental analyses using EDX corroborated the successful incorporation of SDS and AR18 into the PPy films. Thus, EDX spectra, which are included in Figure 5, evidenced the presence of an appreciable concentration of sulfur coming from both SDS and AR18.

**Figure 6:**
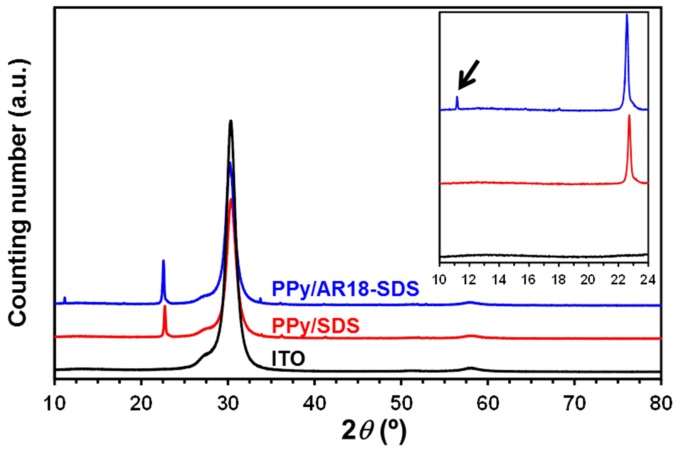
Wide-angle x-ray scattering (WAXS) diagrams were recorded for PPy/SDS and PPy/AR18-SDS supported on ITO and for bare substrate. The inset corresponds to a magnification of the 2*θ* region comprised between 10° and 24°.

WAXS diagrams were recorded for PPy/SDS and PPy/AR18-SDS supported on ITO. Results, including those obtained for ITO control, are displayed in Figure 6. The X-ray diffraction pattern of the ITO substrate suggests a semi-crystalline material in which the crystalline phase was polycrystalline in nature with the predominant peak at 2*θ* = 30.2° and two shoulders at 2*θ* = 26.7° and 58.1°. As it was expected, all these peaks also appeared in the supported CP films. On the other hand, the profile associated with PPy/SDS showed a sharp peak centered at 2*θ* = 22.7°, which was also clearly identified in the spectra recorded for PPy/AR18-SDS. Considering that electropolymerized PPy has a high tendency to cross-link [46,47] and that the X-ray diffraction pattern reported for SDS crystal shows the peak with the strongest intensity at such a position [48,49], the reflection at 2*θ* = 22.7° was attributed to the surfactant. Finally, the PPy/AR18-SDS profile exhibited a weak signal at 2*θ* = 11.2° (Figure 6, inset), which corresponded to the dye, confirming its presence between the CP chains. The intercalation of the dye between the CP chains was observed for PPy combined with Remazol Black B [25].

### 3.3. Optical and Electrochromic Properties

**Figure 7:**
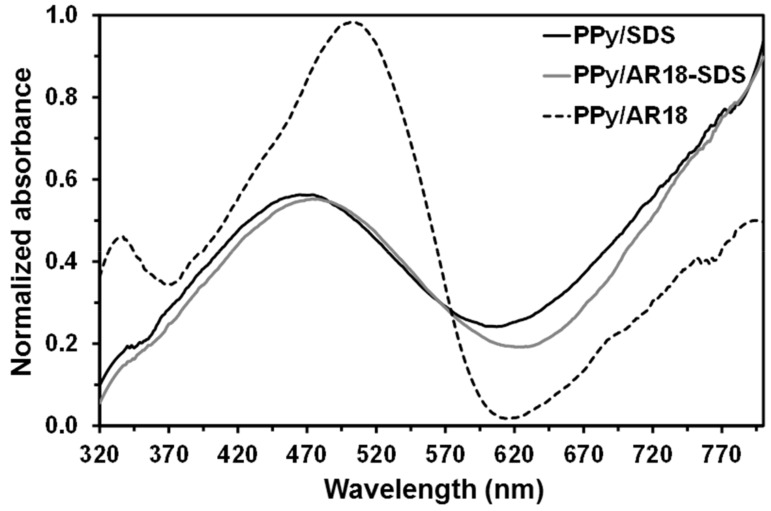
Ultraviolet–visible light (UV–Vis) spectra for PPy/SDS, PPy/AR18-SDS, and PPy/AR18 films.

Figure 7 compares the UV–Vis absorption spectra of PPy/SDS, PPy/AR18-SDS, and PPy/AR18. The wavelength of the absorption maximum (λ_max_), which corresponds to the π–π* transition of the CP backbone, was 38 and 29 cm^−1^ higher for PPy/AR18 than for PPy/SDS and PPy/AR18-SDS, respectively (Table 3). The optical band gap (ε_g_), which was calculated from the onset of the π–π* transition in the absorption spectrum of each film at neutral state, varied between 1.92 and 2.07 eV. Although these ε_g_ values are similar, their variation suggests that the AR18 slightly reduced the π–π* transition energy. This was attributed to the intermolecular π–π stacking interactions between the PPy backbone and the aromatic rings of the dye [50]. Usually, ε_g_ values of CPs are in the range of 1.5–3.0 eV and exhibit several colors and optical changes throughout the visible region [18,51,52,53,54]. CPs with ε_g_ greater than 3.0 eV are known as anodically coloring because they are colorless in the neutral state, while they are absorbing (colored) in the visible region of the oxidized state [55]. In contrast, CPs with ε_g_ lower than 1.5 eV are cathodically coloring materials, which are colored in the neutral state [55]. Accordingly, CPs studied in this work were colored in both neutral and oxidized states. On the other hand, PPy/AR18 showed another absorption band at 336 nm (ε_g_ = 2.8 eV), which was attributed to the azo dye units.

**Figure 8:**
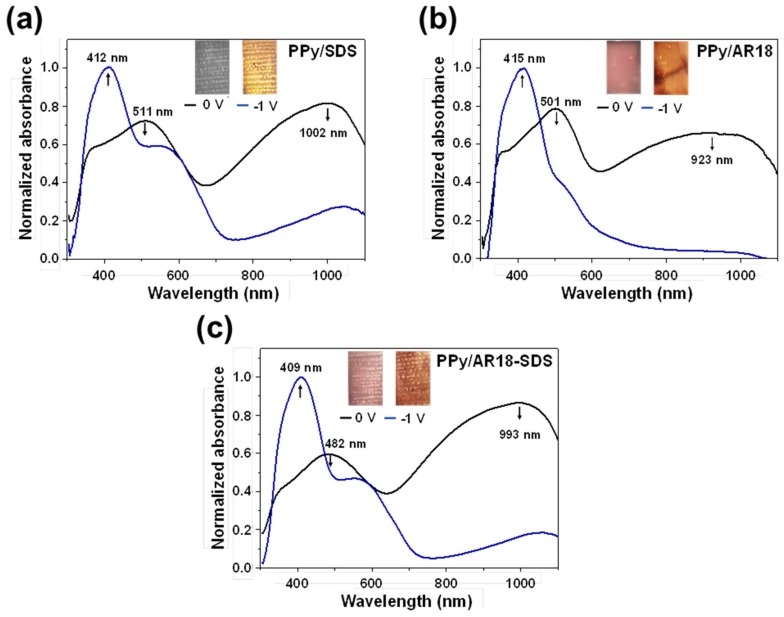
UV–Vis spectra for (**a**) PPy/SDS, (**b**) PPy/AR18, and (**c**) PPy/AR18-SDS films on ITO glass before and after applying −1 V vs. saturated calomel electrode (SCE).

Figure 8a shows the UV–Vis spectra for the PPy/SDS films before and after applying −1 V. Initially, the synthesized film presented a dark-gray color, due to its characteristic absorption band at λ_max_ = 511 nm, which changed to yellow (λ_max_ = 412 nm) when a reduction potential of −1 V vs. SCE was applied for 60 s. This conversion process was totally reversible; hence, after controlled oxidation electrolysis at 1 V vs. SCE, the initial gray color was recovered.

**Figure 9:**
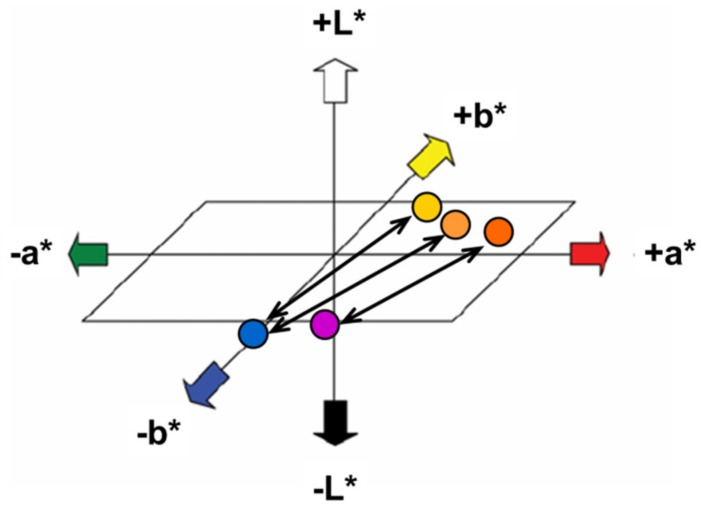
Sketch of a color map showing the change experienced by the three studied systems upon oxidation and reduction.

The same methodology was followed to determine the spectroelectrochemical behavior of PPy/AR18 and PPy/AR18-SDS films. Figure 8b,c show the UV–Vis spectra of initial states and their color changes after applying −1 V vs. SCE. In both cases, a Bordeaux red color was clearly seen due to the presence of the AR18 dye, which showed two absorption bands at λ_max_ = 501 nm and 482 nm. The reduction process of the PPy/AR18 and PPy/AR18-SDS films on ITO led to a change of color in both cases. Hence, an orange coloration appeared in both cases due to the raising of two new absorbance bands at λ_max_ = 415 nm and 409 nm. Additionally, the switching process was also checked by oxidizing the resulting orange films, with the initial state fully recovered. On the other hand, PPy/SDS and PPy/AR18-SDS became blue upon oxidation, even though the color was more intense for the latter than for the former. Color changes associated with redox processes are sketched in Figure 9, which displays an approximate color map for the three studied films. Furthermore, the color of oxidized PPy/AR18 films experienced a change toward dark violet. It is worth noting that such differences in the oxidized films are fully consistent with the optical ε_g_ values (Table 3). Thus, the PPy/SDS and PPy/AR18-SDS films displayed the lightest blue color and the lowest ε_g_ value, whereas PPy/AR18 presented the darkest violet color and the highest ε_g_ value. Inspection of the optical images included in Figure 8 indicates that large globular particles found in PPy/SDS and PPy/AR18-SDS (Figure 5) affected the appearance of the films, even though they were homogeneous and covered the whole ITO surface.

**Figure 10:**
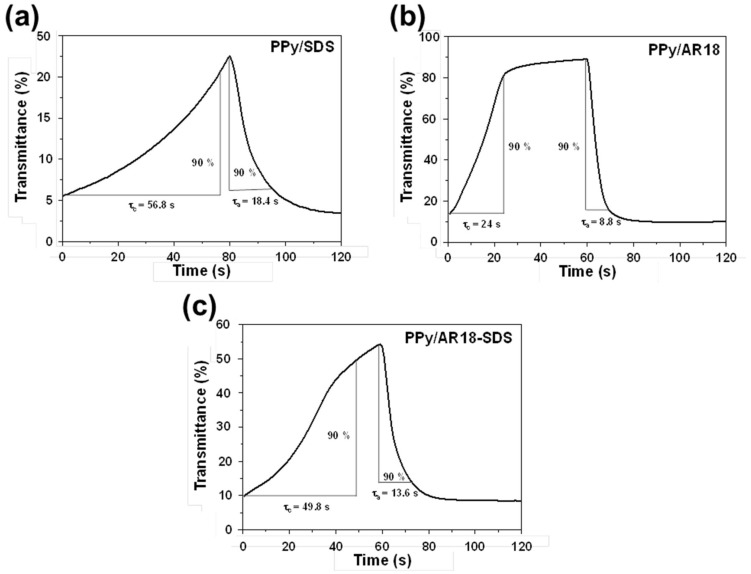
Dynamic change of transmittance after controlled potential electrolysis at −1 V (60 s) and 1 V (60 s) for (**a**) PPy/SDS at λ_max_ = 1002 nm, (**b**) PPy/AR18 at λ_max_ = 923 nm, and (**c**) PPy/AR18-SDS at λ_max_ = 993 nm.

The response time was determined for PPy/AR18-SDS, PPy/AR18, and PPy/SDS films applying a constant voltage of 1.0 V and −1.0 V for 60 s (Figure 10). The response time is defined as the time needed to achieve 90% of the transmittance change when oxidized or reduced (τ_a_ and τ_c_, respectively) [56,57]. The transmittance–time diagram was plotted at the maximum wavelength in each case, λ_max_ = 1002, 923, and 993 nm for PPy/SDS, PPy/AR18, and PPy/AR18-SDS respectively. The response times were larger for PPy/SDS (τ_a_ = 18.4 s and τ_c_ = 56.8 s) and PPy/AR18-SDS (τ_a_ = 13.6 s and τ_c_ = 49.8 s) than for PPy/AR18 (τ_a_ = 8.8 s and τ_c_ = 24.0 s). This observation suggests that the presence of SDS or the interactions between the SDS and AR18 molecules were responsible for the delay in response times.

## 4. Conclusions

The electrochemical properties of azo dye-containing PPy films were compared as a function of the chemical structure of the azo dye, the presence or absence of surfactant in the polymerization medium, and the electrochemical procedure used for the anodic polymerization. The highest electrochemical activity and stability were observed in films produced by CP using a reaction medium that, in addition of the Py monomer, contained a surfactant and the azo dye with the largest number of charged sulfonate groups. More specifically, PPy/AR18-SDS was proven to be a self-stabilizing material, exhibiting increasing electrochemical activity with the number of oxidation–reduction cycles, and well-defined oxidation–reduction reversible processes. The unique behavior of PPy/AR18-SDS, which also presents good optical and electrochromic properties, was attributed to the flexibility and rigidity of SDS and AR18 dopant molecules, respectively, with the former facilitating the entrance of the latter into the polymeric matrix. In summary, the simultaneous incorporation of SDS and AR18 considerably improves the performance of PPy, providing suitable material for use as active layers in optoelectronic devices. This SDS-based approach could be extrapolated to other azo dyes, for example, PPy combined with Remazol Black B, for which electrochromic properties similar to those of PPy/AR18 were reported [25]. Further extension of this work in order to evaluate the stability of PPy/AR18-SDS against environmental conditions (i.e., pH, temperature, UV radiation, etc.) is currently in progress, and results will be reported in due course.

## Figures and Tables

**Table 1:** Polypyrrole (PPy) doped films prepared in this work. The concentration of dopant agent in the reaction medium, as well as the synthetic method, is indicated for each system. The thickness of the films generated by chronopotentiometry (CP) at currents of 1.5 mA/cm^2^ (PPy/ClO_4_^−^ and PPy/SDS; in plain text) or 1.0 mA/cm^2^ (PPy/AR18-SDS, PPy/AR18, PPy/AR1-SDS and PPy/SDS; in italic) is also indicated. CV—cyclic voltammetry; CA—chronoamperometry; SDS—sodium dodecyl sulfate.

System	[ClO_4_^−^] (mM)	[SDS] (mM)	[AR18] (mM)	[AR1] (mM)	Method	*L* (µm)
PPy/ClO_4_^−^	100	-	-	-	CP	5.0 ± 0.3
PPy/SDS	-	100	-	-	CP	4.2 ± 0.2 *5.0* ± *0.3*
PPy/AR18-SDS	-	100	0.5	-	CP, CV, CA	*5.1* ± *0.2*
PPy/AR18	-	-	0.5	-	CP	*6.4* ± *0.2*
PPy/AR1-SDS	-	100	-	0.5	CP	*4.9* ± *0.2*
PPy/AR1	-	-	-	0.5	CP	*6.2* ± *0.2*

**Table 2:** Deposition charge, variation of the electrochemical activity (in %) after 25 and 100 consecutive oxidation and reduction cycles for the systems studied in this work, and Coulombic efficiency after 25 and 100 cycles. For the variation of the electrochemical activity, positive values indicate a loss of redox capacity (i.e., a reduction in electrochemical stability), whereas negative values indicate a gain of electrochemical activity with the number of cycles (i.e., increment in electrochemical stability or self-stabilizing behavior). The Coulombic efficiency is defined as the ratio between the oxidation and reduction charge (Q_ox_/Q_red_).

System	Deposition Charge (C)	Number of Cycles		Coulombic Efficiency	
		25	100	25 Cycles	100 Cycles
PPy/ClO_4_^−^	0.3	16%	-	0.23	-
PPy/SDS	0.3	−4%	-	0.27	-
PPy/SDS	0.2	−6%	10%	0.22	0.19
PPy/AR18	0.2	16%	27%	0.26	0.23
PPy/AR18-SDS	0.2	−29%	−59%	0.33	0.41
PPy/AR1	0.2	3%	-	0.26	-
PPy/AR1-SDS	0.2	−16%	-	0.30	-

**Table 3:** Thickness (*L*), surface roughness (R_q_), and optical properties (λ_max_ and ε_g_) of PPy/SDS, PPy/AR18-SDS, and PPy/AR18 films.

System	*L* (µm)	R_q_ (nm)	λ_max_ (nm)	ε_g_ (eV)
PPy/SDS	4.0	215	465	1.93
PPy/AR18-SDS	5.1	358	474	1.92
PPy/AR18	6.4	45	503	2.07
